# Immunogenicity of Recombinant-Deficient *Lactobacillus casei* with Complementary Plasmid Expressing Alanine Racemase Gene and Core Neutralizing Epitope Antigen against Porcine Epidemic Diarrhea Virus

**DOI:** 10.3390/vaccines9101084

**Published:** 2021-09-26

**Authors:** Fengsai Li, Xiaona Wang, Xiaolong Fan, Ling Sui, Hailin Zhang, Yue Li, Han Zhou, Li Wang, Xinyuan Qiao, Lijie Tang, Yijing Li

**Affiliations:** 1 College of Veterinary Medicine, Northeast Agricultural University, Harbin 150030, China; Yilvwenrou@126.com (F.L.); xiaonawang0319@163.com (X.W.); fxl13796687571@163.com (X.F.); lsseven111@163.com (L.S.); zhanghl0523@163.com (H.Z.); Y1925729473@163.com (Y.L.); zhouhan9659@163.com (H.Z.); wanglicau@163.com (L.W.); qiaoxinyuan@126.com (X.Q.); 2 Heilongjiang Key Laboratory for Animal Disease Control and Pharmaceutical Development, Harbin 150030, China

**Keywords:** *Lactobacillus casei*, PEDV COE antigen, alanine racemase gene, oral mucosal vaccine

## Abstract

Porcine epidemic diarrhea (PED), which is caused by the porcine epidemic diarrhea virus (PEDV), has occurred worldwide and poses a serious threat to the pig industry. Intestine is the main function site of PEDV; therefore, it is important to develop an oral mucosal immunity vaccine against this virus infection. Most traditional plasmid delivery vectors use antibiotic genes as a selective marker, easily leading to antibiotic accumulation and gene contamination. In this study, to explore whether the alanine racemase gene (*Alr*) could be used as a screening marker and develop an efficient oral vaccine against PEDV infection, a recombinant strain was constructed using *Lactobacillus casei* with *Alr* deletion (*L. casei* Δ*Alr W56*) to deliver the *Alr* gene and a core-neutralizing epitope (COE) antigen. This recombinant bacterium efficiently induced secretory immunoglobulin A (SIgA)-based mucosal and immunoglobulin G (IgG)-based humoral immune responses via oral vaccination in mice. Compared to the other strains, the recombinant bacteria were able to grow without the addition of D-alanine, revealing that *Alr* in the plasmid could function normally in defective bacteria. This oral mucosal vaccine would provide a useful strategy to substitute the application of antibiotics in the future and induce efficient immune responses against PEDV infection.

## 1. Introduction

Porcine epidemic diarrhea (PED), characterized by watery diarrhea, vomiting, and dehydration, infects pigs at different ages and is caused by the porcine epidemic diarrhea virus (PEDV) [1]. In recent years, PED has occurred on a large scale in many countries in Asia with a high lethality rate for newborn piglets, leading to significant economic losses to farmers and greatly affecting the development of the pig industry [2]. The PEDV, which belongs to the Coronaviridae family, is an enveloped, single-stranded RNA virus, of which genome comprises five open reading frames encoding an accessory protein and four structural proteins [3]. The spike protein (S) of the PEDV could facilitate cell entry by membrane fusion and induce the production of neutralizing antibodies [4] and contains two regions, S1 and S2, of which S1 is the immunodominant region [5]. It was found that different PEDV strains containing the same epitope region expressed by different hosts have the same reactogenicity as PEDV natural antigens and could induce the body to produce neutralizing antibodies [6]. Thus, the region is used as a potential candidate immunogen against the PEDV [7].

At present, the protective efficacy of traditional commercial vaccines, including transmissible gastroenteritis virus (TGEV)-PEDV dual inactivated vaccines and attenuated vaccines, has gradually decreased, while the incidence of PED has increased every year [8]. PEDV infection has an obvious intestinal tropism; therefore, developing a new oral mucosal vaccine to elicit effective mucosal immune responses against PEDV infection is urgent. However, it is critical to choose a suitable delivery vector to reach the mucosal-immunity-related sites sufficiently and withstand the harsh digestive environment [9]. *Lactic acid bacteria (LAB)* are considered to be safe microorganisms with beneficial effects on the human and animal health [10] by improving the microecological environment of the digestive tract, ensuring the normal physiological state of the host, stimulating specific immune responses and significantly increasing the level of specific and nonspecific antibody in the intestine [11]. *Lactobacillus casei W56* (*Lactobacillus casei W56*) is a member of the LAB group, with a powerful potential as a vaccine delivery vehicle, regulating T-helper cell response and stimulating specific secretory immunoglobulin A (SIgA) secretion for mucosal immunity [12,13]. SIgA plays an important role in the infection of the PEDV, as it is an important component of mucosal immunity [14]. Therefore, oral vaccines would be an effective strategy to induce mucosal immunity and would confer some advantages, such as ease of delivery, safety, and low cost [15].

The traditional biotechnology industry typically uses resistance genes as a screening marker, which is faced with the problem of antibiotic residue contamination and the spread of resistance genes [16]. D-alanine, which is mainly converted from L-alanine by alanine racemase, is an important metabolite of bacteria playing an important role in cell wall synthesis [17]. It was confirmed that a strain with the alanine racemase (*Alr*) gene deletion could result in the incomplete synthesis of the cell wall and the death of the bacteria [18,19,20]. D-alanine is not a common composition of conventional media, and previous studies have shown that its exogenous addition could recover the normal growth of the *Alr* gene deletion bacteria [19,21,22]. This suggests that D-alanine selection may be a promising candidate for the substitution of antibiotics in *Lactobacillus*.

In this study, a genetically engineered strain, *Lactobacillus casei* with *Alr* gene knockout (*L. casei* Δ*Alr W56*), containing a plasmid expressing a core-neutralizing epitope (COE) antigen and the *Alr* gene, was constructed. Its immunogenicity as an oral vaccine was evaluated through the significant levels of anti-PEDV systemic immunoglobulin G (IgG) and mucosal IgA antibody responses in mice. Our results clearly showed that the recombinant strains were effective at inducing anti-PEDV mucosal immune responses.

## 2. Materials and Methods

All animal experiments were approved by the Ethical Committee for Animal Experimentation of Northeast Agricultural University, Harbin, China.

### 2.1. Bacterial Strains, Virus, and Plasmids

*L. casei* Δ*Alr W56* was constructed in our laboratory and grown anaerobically in a de Man, Rogosa, and Sharpe (MRS) broth supplemented with D-alanine at 37 °C without shaking. PEDV LJB/15 strain was dissociated from clinical samples by our laboratory and propagated in porcine small intestinal epithelial cells (IPECs) at 37 °C with 5% CO_2_. The constitutive expression plasmid pPG-T7g10-PPT, with a hyper constitutive expression promoter, T7g10 enhancer, pgsA anchor, and rrnBT1T2 terminator, was constructed in our laboratory. The details of all plasmids and primers used in the study are listed in Table 1 and Table 2.

### 2.2. Construction of Recombinant L. casei Strains

**Figure 1:**
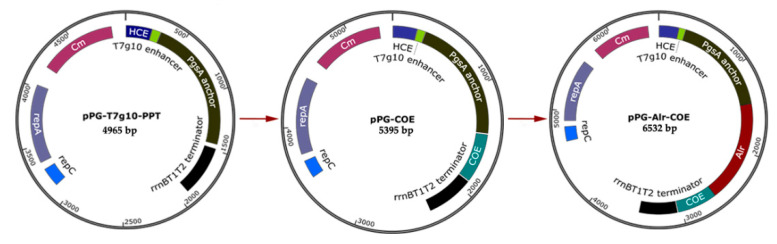
Schematic diagram of the construction recombinant plasmids. Plasmid pPG-Alr-COE was constructed as follows. The gene encoding COE was amplified from the plasmid preserved in our laboratory with *Sac* I and *Apa* I digestion and inserted into the corresponding expression plasmid pPG-T7g10-PPT, yielding the recombinant plasmid pPG-COE. Then, the gene fragment *Alr* obtained from the genome of *Lactobacillus casei* (*L. casei*) *W56* was inserted into the plasmid pPG-COE, producing the recombinant plasmid pPG-Alr-COE.

A schematic diagram of the recombinant plasmid construction is shown in Figure 1. Briefly, the gene encoding the COE antigen of PEDV was amplified with the plasmid pMD18T-COE as the template. The gene fragment *Alr* was obtained from the genome of *L. casei W56*. The COE gene was then inserted at the *Sac* I and *Apa* I sites of the corresponding expression vector pPG-T7g10-PPT constructed by Song et al. [23], generating the recombinant plasmid pPG-COE. Subsequently, *Alr* was cloned into the recombinant vector pPG-COE, resulting in the plasmid pPG-Alr-COE. All recombinant plasmids were assessed by polymerase chain reaction (PCR) and sequencing.

To construct the recombinant strains, *L. casei* Δ*Alr W56* competent cells were prepared according to an electroporation method described previously, resulting in the recombinant strains pPG-COE/Δ*Alr W56*, pPG-Alr-COE/Δ*Alr W56*, and pPG-T7g10-PPT/Δ*Alr W56* [24]. Briefly, 500 ng of recombinant plasmids were gently mixed with 200 μL of the competent cells, after which the mixture was transferred into a pre-cooled Gene Pulser™ (Bio-Rad, Hercules, CA, USA) disposable cuvette (inter-electrode distance, 0.2 cm) and exposed to a single electric pulse (2.2 V, 200 Ω, 25 μF) with a Gene Pulser™ (Bio-Rad). The strains were cultured in MRS plates with D-alanine at 37 °C for 24 h. The plasmids were extracted, and the genes *COE* and *Alr* were identified by PCR confirmation and sequencing. These strains were constructed successfully and streaked on chloramphenicol MRS plates with or without D-alanine to detect the demand situation.

### 2.3. Identification of the Expression of the Protein of Interest

To evaluate the expression of the protein of interest by the recombinant strains, pPG-COE/ΔAlr W56, pPG-Alr-COE/Δ*Alr W56*, pPG-T7g10-PPT/Δ*Alr W56*, and Δ*Alr W56* were grown overnight in MRS broth with or without D-alanine, harvested by centrifugation at 10,000× *g* for 2 min and washed twice with sterile phosphate-buffered saline (PBS). The supernatants were lysed and centrifuged using a Mini-Beadbeater (BioSpec, Bartlesville, OK, USA) and separated in a 12% gel by sodium dodecyl sulfate-polyacrylamide gel electrophoresis (SDS-PAGE) for the western blot assay. The proteins were then transferred to a polyvinylidene difluoride membrane (Millipore, Milford, MA, USA). Immunoblotting was performed using a mouse anti-Flag antibody (1:1000) as the primary antibody and horseradish peroxidase (HRP)-conjugated goat anti-mouse IgG antibody (1:5000) as the secondary antibody (Sigma-Aldrich, St. Louis, MO, USA). The results were visualized using a chemiluminescent substrate reagent (Thermo Fisher Scientific, Durham, NC, USA) according to the manufacturer’s instructions. 

### 2.4. Immunization

All the experimental and animal management procedures were approved by the Institutional Committee of the Northeast Agricultural University for the Animal Experiments (2016NEFU-315; 13 April 2017), Harbin, China. To assess the immunogenicity of recombinant strains applied as an oral vaccine, four-week-old specific-pathogen-free (SPF) female BALB/c mice (*n* = 120) were purchased from Liaoning Changsheng Biotechnology Co., Ltd. (Benxi, China) and maintained under SPF conditions for one week with free access to a standard chow diet and water in accordance with institutional guid/elines. Prior to oral administration, the recombinant strains were cultured overnight in an MRS medium with or without D-alanine, washed with sterile PBS and resuspended in PBS at a concentration of 10^10^ colony forming units (CFU) CFU mL^−1^. At the indicated time points, three mice were randomly selected from each of the 4 groups (each of the 4 groups had 30 mice each). The mice were immunized with 200 μL of the whole volume live recombinant *L. casei* by oral gavage, once for 3 consecutive days (days 1, 2, and 3) and boosted twice at 2 week intervals (days 14, 15, and 16 and 28, 29, and 30).

### 2.5. Sample Collection

The immunized mice were randomly selected three in the different groups and collected the samples, respectively. The mice were sacrificed, and the sera and intestinal mucusal were collected from the first immunized mice on days 0, 7, 14, 21, 28, 35, 40, 42, 49, 56, 63, and 70 and stored at −40 °C. Intestinal mucusal was scraped with a glass slide for detection after rinsing with sterile PBS. Vaginal and nasal fluid were collected by aspirating and discarding repeatedly with 500 µL sterile PBS to stimulate the nose of mice for sample collection and stored at −40 °C until analysis. Fecal samples were collected at different timepoints, used for detecting the SIgA antibody and treated according to methods described previously with slight modifications [25]. Briefly, a 0.1 g fecal pellet was suspended in 400 µL of PBS containing 1 mmoL L^−1^ phenylmethylsulfonyl fluoride (Sigma-Aldrich) and 1% bovine serum albumin, incubated at 4 °C for 16 h and centrifuged at 10,000× *g* for 5 min, after which the supernatants were stored at −40 °C until use.

### 2.6. Enzyme-Linked Immunosorbent Assay (ELISA) Analysis

To determine the levels of the anti-PEDV-specific IgG antibody in the sera and the SIgA antibody in the fecal, intestinal mucusal, vaginal, and nasal fluids, the samples were collected from the immunized mice at the corresponding timepoints and measured using ELISA. The PEDV propagated on IPECs were coated on polystyrene microtiter plates overnight at 4 °C, and the culture of IPECs was used as a negative control for the antigen. After blocking with 5% skimmed milk and washing three times with 0.1% PBS Tween 20 (PBST), the collected samples were serially diluted in PBS (fecal, intestinal mucusal, vaginal, and nasal fluid samples) or skimmed milk (sera), added in triplicate and incubated at 37 °C for 2 h. Next, an HRP-conjugated goat anti-mouse IgG or IgA antibody (Invitrogen, Carlsbad, CA, USA) was added to each well (1:5000 or 1:2000, respectively) and incubated at 37 °C for 1 h. The color was detected using the o-phenylenediamine dihydrochloride substrate (Sigma-Aldrich) and measured at an absorbance of 490 nm. To detect the level of cytokines, sera samples were collected from the immunized mice and subjected to ELISA according to the manufacturer’s instructions (Invitrogen, USA) on 40 d post-immunization. The assays included IL-4, IFN-γ, and IL-17. The detection ranges of these cytokine detection kits were as following: IFN-γ (47–300 pg/mL), IL-4 (0.63–40 pg/mL), and IL-17 (7.8–500 pg/mL).

### 2.7. Neutralizing Activity of the Samples

To determine the neutralizing activity of the sera or intestinal mucusal antibodies obtained from the mice immunized with pPG-Alr-COE/Δ*Alr W56*, 50 µL 2 × serial diluted samples were collected on 40 d post-immunization, mixed with 50 µL of PEDV passages with 200 50% tissue culture infective dose (TCID_50_) in IPECs and incubated at 37 °C for 1 h. The mixture was then incubated to IPECs monolayers in 96-well plates. PEDV-specific cytopathic effects (CPEs) were observed with incubation at 37 °C in a 5% CO_2_ atmosphere for 3 days. The Reed–Muench statistical method was applied for analyzing the data [26].

### 2.8. Statistical Analysis

GraphPad Prism (version 7.0; GraphPad Software, San Diego, CA, USA) was used to perform the statistical analyses. Tukey’s multiple comparison test was used to analyze the differences among the control groups. *p* < 0.05 was considered statistically significant, and *p* < 0.01 was considered highly significant.

## 3. Results

All animal experiments were approved by the Ethical Committee for Animal Experimentation of Northeast Agricultural University, Harbin, China.

### 3.1. Construction of Recombinant Lactobacillus Casei Strains and Demand Verification

**Figure 2:**
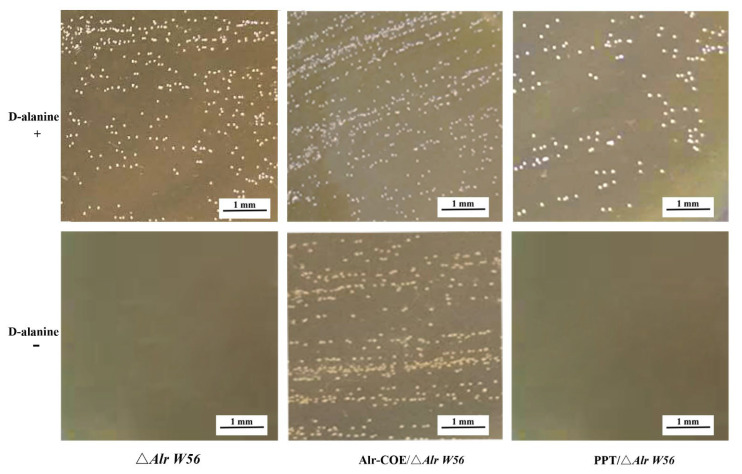
The D-alanine demand identification of recombinant *L. casei* strain pPG-Alr-COE/Δ*Alr W56*. The recombinant strains pPG-Alr-COE/Δ*Alr W56*, pPG-T7g10-PPT/Δ*Alr W56*, and Δ*Alr W56* were streaked on de Man-Rogosa-Sharpe (MRS) plates to detect the demand of D-alanine. “+” and “−” represent MRS plates with and without D-alanine, respectively; Alr-COE/Δ*Alr W56*, pPG-Alr-COE/Δ*Alr W56*; PPT/Δ*Alr W56*, pPG-T7g10-PPT/Δ*Alr W56*.

For constructing the recombinant strains, the plasmid pPG-Alr-COE was transferred into *L. casei* Δ*Alr W56* competent cells, then purified and identified by standard PCR and sequencing. Subsequently, the requirement for D-alanine was verified. As shown in Figure 2, all recombinant strains were able to grow on an MRS agar medium in the presence of D-alanine, while only the strain pPG-Alr-COE/Δ*Alr W56* was able to grow in a medium without D-alanine. These results indicated that the recombinant strains were successfully constructed, and the *Alr* gene in the plasmid could play a similar role to that in the *L. casei W56* genome. 

### 3.2. Detection of Expression of the Protein of Interest

**Figure 3:**
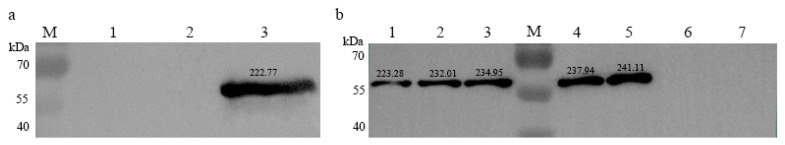
Expression of the protein of interest identified and stable inheritance by western blot detection. (**a**) Expression of interest proteins Alr-COE by the recombinant *L. casei* was detected by western blot with an anti-flag monolconal antibody. Numbers 1–3 represent the strains pPG-T7g10-PPT/Δ*Alr W56,* Δ*Alr W56* and pPG-Alr-COE/Δ*Alr W56*, respectively. (**b**) Western blot analysis certificated that the expression of interest proteins Alr-COE by *L. casei* pPG-Alr-COE/Δ*Alr W56* was stable heredity within 50 generations. Numbers 1–5 represent *L. casei* pPG-Alr-COE/ΔAlr W56 strains 10th to 50th generation with every 10-generation protein expression, respectively; numbers 6–7 represent the strains pPG-T7g10-PPT/Δ*Alr W56* and Δ*Alr W56*, respectively. The datas shown in the figture were densitometry readings/intensity ratio.

To identify the expression of the protein of interest in the recombinant strains, pPG-Alr-COE/Δ*Alr W56*, pPG-T7g10-PPT/Δ*Alr W56*, and Δ*Alr W56* were cultured overnight in MRS media with or without D-alanine, and cell lysates were analyzed by western blot assay with an anti-flag monoclonal antibody as the primary antibody (The original results of western blot were shown in the Supplementary Material, Figure S1 and Figure S2). As shown in Figure 3, the expected immunoblot band of Alr-COE expressed by pPG-Alr-COE/Δ*Alr W56* was approximately 55 kDa, consistent with the predicted molecular weight, but no bands were observed in pPG-T7g10-PPT/Δ*Alr W56* and Δ*Alr W56*. The hereditary stability of the protein expressed by recombinant *L. casei* strains were evaluated in more than 50 generations with detection performed every 10 generations, and it was found to be stably inherited. 

### 3.3. SIgA Levels Induced by Oral Recombinant Strains

**Figure 4:**
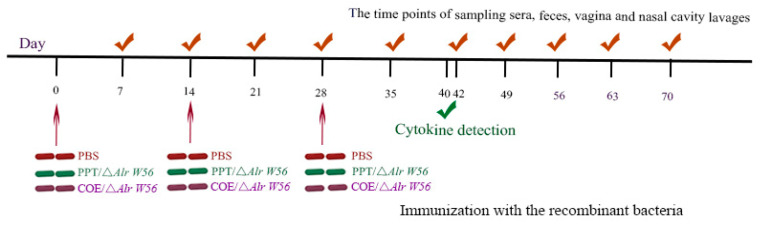
Procedure of oral immunization and sampling. Fecal, vaginal fluid, nasal fluid, sera, and intestinal mucusal are shown with the means of red checks, and green checks indicate the level of cytokine detection. Red strains represent the time points used for the administration of phosphate-buffered saline (PBS); green strains indicate the points of administration empty carrier *L. casei* pPG-T7g10-PPT/Δ*Alr W56*; purple strains stand for the time points used for the administration of recombinant *L. casei* pPG-Alr-COE/Δ*Alr W56*. The groups of mice were orally immunized with recombinant *L. casei* on three consecutive days (days 1, 2, and 3), a second immunization was administered on days 14, 15, and 16, and a third immunization was administered on days 28, 29, and 30. Then, sample collection was gathered at different time points with the first post-immunization.

**Figure 5:**
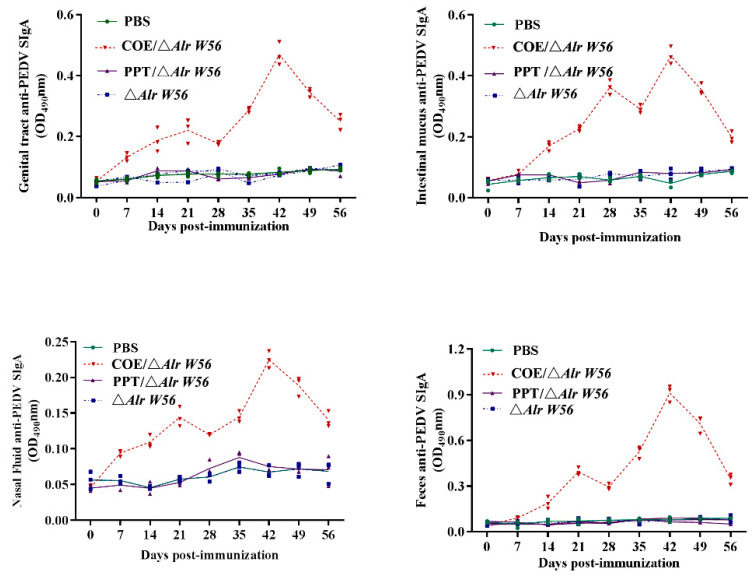
The levels of anti-PEDV-specific IgA in immunized mice. The mice were divided into four groups with 30 in every group. Anti-PEDV specific sIgA levels in the fecal, vaginal fluid, intestinal mucosal, and nasal fluid post-immunization with recombinant strains were detected by indirect enzyme-linked immunosorbent assay (ELISA). The samples from each group were analyzed (neat solution) as one sample in triplicate.

To analyze the level of SIgA induced by oral recombinant strain pPG-Alr-COE/Δ*Alr W56*, fecal, vaginal fluid, intestinal mucosal, and nasal fluid samples were collected at different time points, using the BALB/c mice and PEDV LJB/15 strain as the model and the antigen, respectively, detected by indirect ELISA. The immunization procedure is shown in Figure 4. Three rounds of immunization were conducted, with each lasting three days with an interval of 14 days. In Figure 5, it could be observed that anti-PEDV-specific SIgA antibodies were detected at a high-level 7 d post-immunization, significantly increased after booster immunization and peaked at 42 d post-immunization. The level of anti-PEDV-specific mucosal sIgA in mouse fecal, vaginal fluid, intestinal mucosal, and nasal fluids orally immunized with pPG-Alr-COE/Δ*Alr W56* was significantly higher (*p* < 0.01) than those in the PBS, pPG-T7g10-PPT/Δ*Alr W56*, and Δ*Alr W56* groups. No significant difference (*p* > 0.05) was observed in the PBS, pPG-T7g10-PPT/Δ*Alr W56*, and Δ*Alr W56* groups pre- and post-immunization. These results showed that the recombinant bacteria could effectively induce mucosal immunity. 

### 3.4. IgG Levels and Neutralizing Antibody Activity Induced by Oral Recombinant Strains

**Figure 6:**
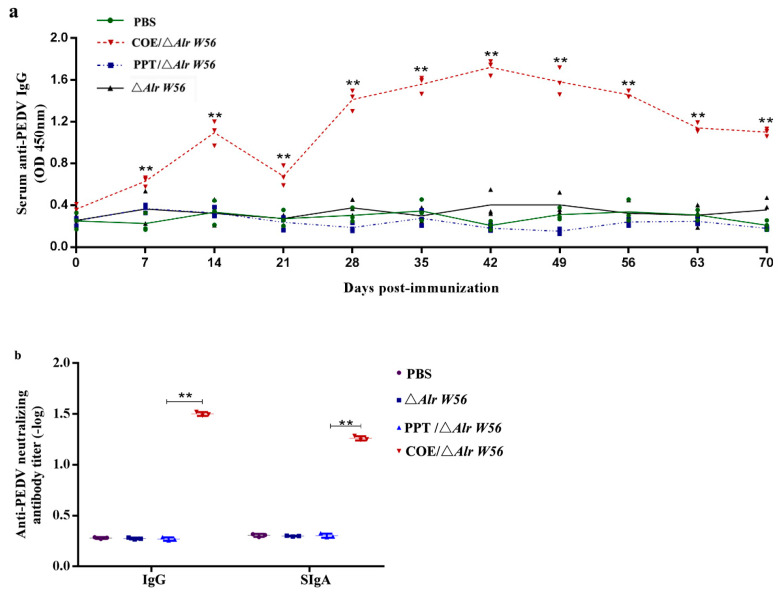
Levels of the anti-PEDV-specific immunoglobulin G (IgG) antibody (**a**) and the anti-PEDV-neutralizing antibody titer (**b**) in mice post-immunization. The mice were divided into four groups with 30 in every group. The results were ascertained by measuring the anti-PEDV IgG antibody and the anti-PEDV-neutralizing antibody titer with 2 × serial dilution in sera (dilution in 5% skimmed milk) and intestinal mucus (dilution in PBS) from immunized mice by indirect ELISA (**a**) using the PEDV as the coating antigen and virus neutralization experiment (**b**). ** *p* < 0.01 compared to the control groups.

To measure the IgG level induced by oral recombinant strain pPG-Alr-COE/Δ*Alr W56*, sera were collected from the first immunized mice on days 0, 7, 14, 21, 28, 35, 42, 49, 63, and 70 (Figure 6a). Our results showed that since the 7th day with the initial immunization, the recombinant strain pPG-Alr-COE/Δ*Alr W56* group produced specific antibodies. After boosting immunity, the level of sera IgG significantly increased and reached its peak 42 d post-immunity. There was no obvious difference among these groups before the primary immunization, and the pPG-Alr-COE/Δ*Alr W56* group showed a significantly higher level of anti-PEDV-specific IgG antibody than the control groups (PBS, pPG-T7g10-PPT/Δ*Alr W56*, and Δ*Alr W56* groups; *p* < 0.01). Expectedly, no significant elicitation of the anti-PEDV IgG antibody was observed in the control groups of mice, including PBS, pPG-T7g10-PPT/Δ*Alr W56*, and Δ*Alr W56*. Moreover, a virus neutralization assay was performed to assess the PEDV-neutralizing activity of the sera and intestinal mucosal antibodies stimulated in the immunized mice 40 d post-immunization, as shown in Figure 6b. The results showed that the antibody activity in mice induced by pPG-Alr-COE/Δ*Alr W56* exhibited a stronger anti-PEDV-neutralizing activity than that in mice orally administered with PBS, pPG-T7g10-PPT/Δ*Alr W56*, and Δ*Alr W56*. As expected, the group pPG-Alr-COE/Δ*Alr W56* expressing the COE antigen could effectively arouse an anti-PEDV immune response in vivo.

### 3.5. Production of Cytokines Excited by Oral Recombinant Strains

**Figure 7:**
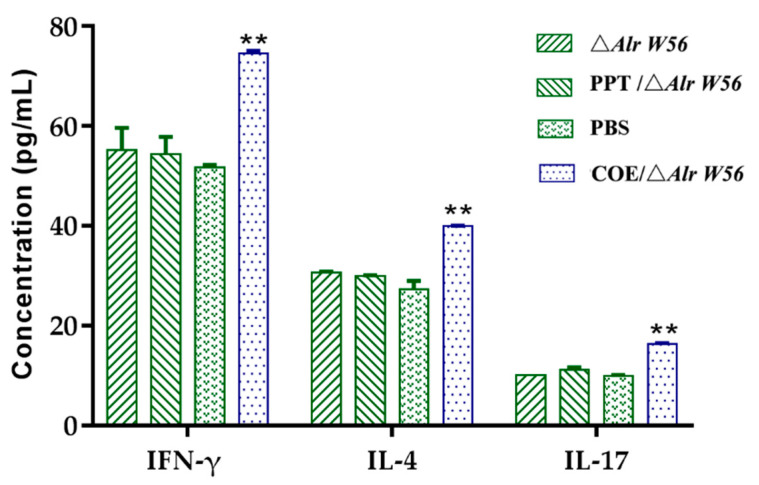
Determination of serum cytokines produced by the mice (n = 3/group) orally administered with pPG-Alr-COE/Δ*Alr W56*, pPG-T7g10-PPT/Δ*Alr W56*, Δ*Alr W56*, or PBS separately. Samples were collected 10 days with the third post-immunization (40 days post the first immunization) and tested with a commercial kit. ** *p* < 0.01 compared with control groups.

To compare cellular and humoral immunity excited by the immunized mice oral recombinant bacteria, the cytokines were detected using a standardized kit. Sera were collected through retro-orbital bleed from four groups of mice (three in each group) 40 d post-first immunization, to detect the cytokine IL-4 and IFN-γ, as shown in Figure 7. The results showed that the levels of secreted cytokine induced by pPG-Alr-COE/Δ*Alr W56* was higher than those induced by PBS, pPG-T7g10-PPT/Δ*Alr W56*, and Δ*Alr W56* groups (*p* < 0.05), and among these three, no significant difference was observed (*p* > 0.05). The ratio of IL-4 (Th2-type response) to IFN-γ (Th1-type response) is usually applied to analyze the immune level of Th1 or Th2. In the current study, the IL-4/IFN-γ ratio was 1.12 (Figure 7), revealing that the strain pPG-Alr-COE/Δ*Alr W56* could induce both Th2 and Th1 immune responses but was focused on Th2. These results illustrated that the recombinant strains can well induce humoral and cellular immune responses.

## 4. Discussion

In recent years, the diarrhea epidemic dominated by the PEDV has occurred in Taiwan, Italy, Germany, and America, of which the mortality reached 100%, causing severe economic losses in the pig industry [27,28]. In China, it was reported that the vaccination failure and the high death rate in the 2010 Chinese outbreak were due to the changes in the antigenicity of the PEDV based on high mutation rates [29]. Studies have shown that the COE (499–638 aa) of the PEDV S protein can induce neutralizing antibodies to protect against PEDV infection [30]. Therefore, it might be a useful strategy to construct a vaccine with the S-protein-neutralizing epitope of the PEDV to combat virus infection. In the study, the gene encoding COE was preserved in our laboratory, isolated from a PEDV outbreak in the area, with a high homology of new strains in recent years at home and abroad, i.e., more than 98%.

PEDV infection mainly occurs on the mucosal surface, and the mucosal immune system is the first barrier against viral infections [31]. In piglets, SIgA is the key factor against PEDV infection, as it can protect epithelial cells from damage by neutralizing the PEDV in the small intestine, thereby playing an important role in the passive immunity of animal bodies [32]. Thus, developing a mucosal vaccine based on PEDV infection mainly through the mucosal pathway and elucidating the important role of SIgA antibodies against the disease could contribute to effectively stimulating the mucosal immune system to produce local and systemic immune responses. 

*Lactobacillus* is often applied as a vaccine delivery vehicle. It owns many advantages, such as the resistance to the harsh GI environment, the powerful capacity for intestinal colonization, and the nonspecific immunoadjuvant effect [33,34,35]. Many studies have focused on using *Lactobacillus* as a live carrier to develop oral mucosal vaccines against pathogens owing to its safeness and nonspecific adjuvant effect [36,37]. In 2015, Cui et al. introduced the G protein of carp virus (SVCV) into a *Lactobacillus* live vector vaccine to improve the level of immune response and achieve a good immune protection rate [38]. Ma et al. applied the intestinal M cells- and DCs-targeting gene combined with the COE antigen of the PEDV expressed in genetically engineered *Lactobacillus* to orally immunized mice, efficiently inducing anti-PEDV mucosal, humoral, and cellular immune [39]. *L. casei W56* as one of *Lactobacillus* could attack and colonize in intestinal mucosa and has bile tolerance and acid resistance, thus serving as a promising candidate to deliver the antigenic protein to the mucosa [40]. Qiao et al. used *L. casei* as an antigen delivery vehicle to immunize mice and obtained a good immune effect [41]. Wang et al. utilized *L. casei* as an antigen delivery vector to express Rabbit hemorrhagic disease virus (RHDV) capsid protein VP60(VP1)-fused eGFP, and the results indicated that the oral administration of this probiotic vaccine could stimulate SIgA-based mucosal and IgG-based humoral immune responses in rabbits [42]. In this study, we used *L. casei* Δ*Alr W56* to deliver the PEDV S protein-neutralizing epitope COE fragment and the *Alr* gene and to develop an oral mucosal vaccine against the PEDV and be used as a screen marker in the future. In traditional molecular biology, antibiotic genes are often used as a selectable marker of the plasmid. In addition, although the widespread use of antibiotics is indispensable for human health and social development, the harm has become increasingly significant. To address this, a new selective marker should be used. We have found that deleting the *Alr* gene may cause bacterial death; in contrast, strain growth is maintained following the exogenous addition of D-alanine [43,44]. Therefore, *Alr* is considered as a candidate to replace the use of antibiotic genes [45]. We ligated the *Alr* gene into the recombinant plasmid pPG-COE and electrotransformed it into *L. casei* Δ*Alr W56*. The results revealed that the recombinant bacteria are able to grow in an MRS broth without the exogenous addition of D-alanine, while other strains are not, indicating that the *Alr* gene in the plasmid could complement deficient strains to be applied as a selective marker in the future. In this study, we used the genetically engineered pPG-Alr-COE/Δ*Alr W56* as an oral vaccine to immunized mice, eliciting a humoral immune response against PEDV. This was proved by the significant levels of virus-neutralizing antibodies and anti-PEDV serum IgG in mice orally immunized with pPG-Alr-COE/Δ*Alr W56*, compared to the levels in the PBS, pPG-T7g10-PPT/Δ*Alr W56*, and Δ*Alr W56* groups, demonstrating good immunogenicity and a potential vaccine strategy against PEDV infection. 

Mucosal immunity plays an important role in preventing viral diarrheal diseases, and sIgA antibodies are a good way for piglets to acquire immunoprotection [46]. In this study, BALB/c mice were used as an animal model to evaluate the immunogenicity of pPG-Alr-COE/Δ*Alr W56*, inducing the mucosal and systemic responses against PEDV. An antigen-specific SIgA antibody in the feces, intestinal mucus, and vaginal and nasal fluid was detected by ELISA post-oral immunization. Our data revealed that there was a noticeable increase of the anti-PEDV-specific mucosal SIgA antibody in these samples induced in the mice orally immunized with pPG-Alr-COE/Δ*Alr W56* after a second booster immunization. Thus, the genetically engineered strain pPG-Alr-COE/Δ*Alr W56* as an oral vaccine can effectively induce antigen-specific mucosal response against PEDV infection.

T-helper cells are divided into two major subsets, Th1 and Th2 cells. LAB can induce local mucosal immune responses in the intestine, and an appropriate dose can promote Th0 differentiation and boost B lymphocytes to produce SIgA [47]. Cytokines are the main factors that determine the differentiation direction of Th0 cells. IFN-γ promotes the differentiation of Th1-type cells, and IL-4 and IL-17 promote the differentiation of Th2- and Th17-type cells, respectively. The rise of the levels of the representative cytokines IFN-γ, IL-4, and IL-17 suggests that the body induces cellular and humoral immune response [25,48]. In this study, we tested the cytokines IFN-γ and IL-4. Compared with those in the PBS, pPG-T7g10-PPT/Δ*Alr W56*, and Δ*Alr W56* groups, the levels of the cytokine oral recombinant strain pPG-Alr-COE/Δ*Alr W56* significantly increased, indicating that oral recombinant strains stimulate mice to produce Th1- and Th2-cell type immunity. The IL-4/IFN-γ ratios in the experimental groups were higher than that of the control group, revealing that the cellular immunity induced by oral recombinant bacteria pPG-Alr-COE/Δ*Alr W56* was mainly Th2-cell-based immunity. Therefore, oral immunization with genetically engineered pPG-Alr-COE/Δ*Alr W56* may provide effective protection against PEDV infection.

In addition, our results showed that the recombinant pPG-Alr-COE/Δ*Alr W56* oral vaccine (with an *Alr* gene in the plasmid) exhibited excellent immunogenicity and can grow without D-alanine. It is worth mentioning that the *Alr* gene was used as a selective marker in *L. casei* Δ*Alr W56* to avoid the main disadvantage of traditional plasmid expression systems that employ antibiotic resistance genes as a selective pressure for genetically engineered bacteria [49,50]. Moreover, pPG-T7g10-PPT was constructed with a constitutive expression plasmid in our laboratory containing a pgsA-derived anchoring matrix from *Bacillus subtilis* [51]; thus, the fusion proteins were displayed on the bacterial surface, eliciting good immunogenicity.

In conclusion, an oral mucosal vaccine pPG-Alr-COE/Δ*Alr W56* was constructed in this study, providing a candidate replacement for antibiotic genes used as selection marker in the future. We confirmed that the genetically engineered strain pPG-Alr-COE/Δ*Alr W56* could efficiently induce mucosal, humoral, and cellular immune responses against the PEDV, suggesting that it may be a promising strategy for vaccine development against PEDV infection. In the future, the immunization and challenge protection experiments would be conducted on the host animal pigs to further evaluate the vaccine value of recombinant strains and lay a foundation for the preparation of new vaccines.

## Figures and Tables

**Table 1:** Details of plasmids used in this study.

Plasmids	Relevant Characteristics	Description/Reference
pMD19-Ts-COE	3158bp; *Amp^r^*; the amplified DNA fragment core-neutralizing epitope (COE) was inserted into pMD19-Ts	This study
pMD19-Ts	2692 bp; *Amp^r^*	Takara (Dalian, China)
pMD19-Ts-Alr	3844 bp; *Amp^r^;* the amplified DNA fragment *Alr* was inserted into pMD19-Ts	This study
pPG-T7g10-PPT	4965 bp; *Cm^r^*; HCE promoter; PgsA anchor; constitutive expression plasmid	Preserved in our Lab.
pPG-COE	5395 bp; *Cm^r^*; COE was inserted into pPG-T7g10-PPT	This study
pPG-Alr-COE	6532 bp; *Cm^r^*; *Alr* was inserted into pPG-COE	This study

*Amp^r^*: ampicillin resistance; *Cm^r^*: chloramphenicol resistance.

**Table 2:** Details of primers used in this study.

Gene	ID	Primers Sequences
COE	COE-F COE-R	CGAGCTCATGGGTACC**GATTATAAGGATGACGATGACAAG**TAGAAACCTTCTGAGTCATG TAGGGCCCGTAATCAACTCACCCTTTGT
*Alr*	alr-F alr-R	CGAGCTCCTGAACGTGACGATCGGTAA TAGGTACCTCAATCGACCGGATTCACGC

Restriction enzyme recognition sites used for cloning are underlined. Flag tags were shown in bold.

## Data Availability

Not applicable.

## Supplementary Materials

The following are available online at https://www.mdpi.com/2076-393X/9/10/1084/s1, Figure S1: Expression of the protein of interest identified by western blot detection; Figure S2: The stable expression of the protein of interest identified by western blot detection.
