# Correction: Li et al. Immunogenicity of Recombinant-Deficient *Lactobacillus casei* with Complementary Plasmid Expressing Alanine Racemase Gene and Core Neutralizing Epitope Antigen against Porcine Epidemic Diarrhea Virus. *Vaccines* 2021, *9*, 1084

**DOI:** 10.3390/vaccines11020388

**Published:** 2023-02-08

**Authors:** Fengsai Li, Xiaona Wang, Xiaolong Fan, Ling Sui, Hailin Zhang, Yue Li, Han Zhou, Li Wang, Xinyuan Qiao, Lijie Tang, Yijing Li

**Affiliations:** 1 College of Veterinary Medicine, Northeast Agricultural University, Harbin 150030, China; 2 Heilongjiang Key Laboratory for Animal Disease Control and Pharmaceutical Development, Harbin 150030, China

In the original publication [1], there was an error in Figure 2 as published. In Figure 2, due to too many pictures in the data processing process, some pictures were repeated.

The corrected Figure 2 appears below.

The authors state that the scientific conclusions are unaffected. This correction was approved by the Academic Editor. The original publication has also been updated.

## Figures and Tables

**Figure 2:**
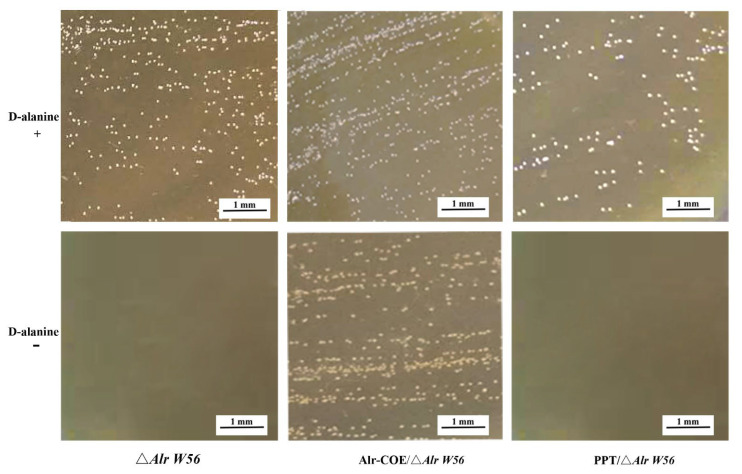
The D-alanine demand identification of recombinant *L. casei* strain pPG-Alr-COE/Δ*Alr W56*. The recombinant strains pPG-Alr-COE/Δ*Alr W56*, pPG-T7g10-PPT/Δ*Alr W56*, and Δ*Alr W56* were streaked on de Man-Rogosa-Sharpe (MRS) plates to detect the demand of D-alanine. “+” and “−” represent MRS plates with and without D-alanine, respectively; Alr-COE/Δ*Alr W56*, pPG-Alr-COE/Δ*Alr W56*; PPT/Δ*Alr W56*, pPG-T7g10-PPT/Δ*Alr W56*.
