# Supplementary material for: Immunogenicity of Recombinant-Deficient Lactobacillus casei with Complementary Plasmid Expressing Alanine Racemase Gene and Core Neutralizing Epitope Antigen against Porcine Epidemic Diarrhea Virus
Source: Vaccines (Basel). 2021 Sep 26;9(10):1084. doi: 10.3390/vaccines9101084 (PMC8537014; doi:10.3390/vaccines9101084)
Supplement: Supplementary file 1 [file vaccines-09-01084-s001.zip › vaccines-1355793 Supplementary materials.docx]

Supplementary Material

Here are the original images for Blots/Gels, and no cropping and adjustments have been made to the icons.


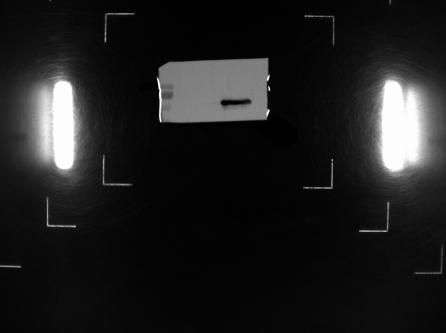


**Figure S1.** Expression of the protein of interest identified by western blot detection. The picture was the original images for Blots. 1-3 lines represent △*Alr W56*, pPG-T7g10-PPT/△*Alr W56,* and pPG-Alr-COE/△*Alr W56* respectively. The densitometry readings/intensity ratio of band was 222.77.


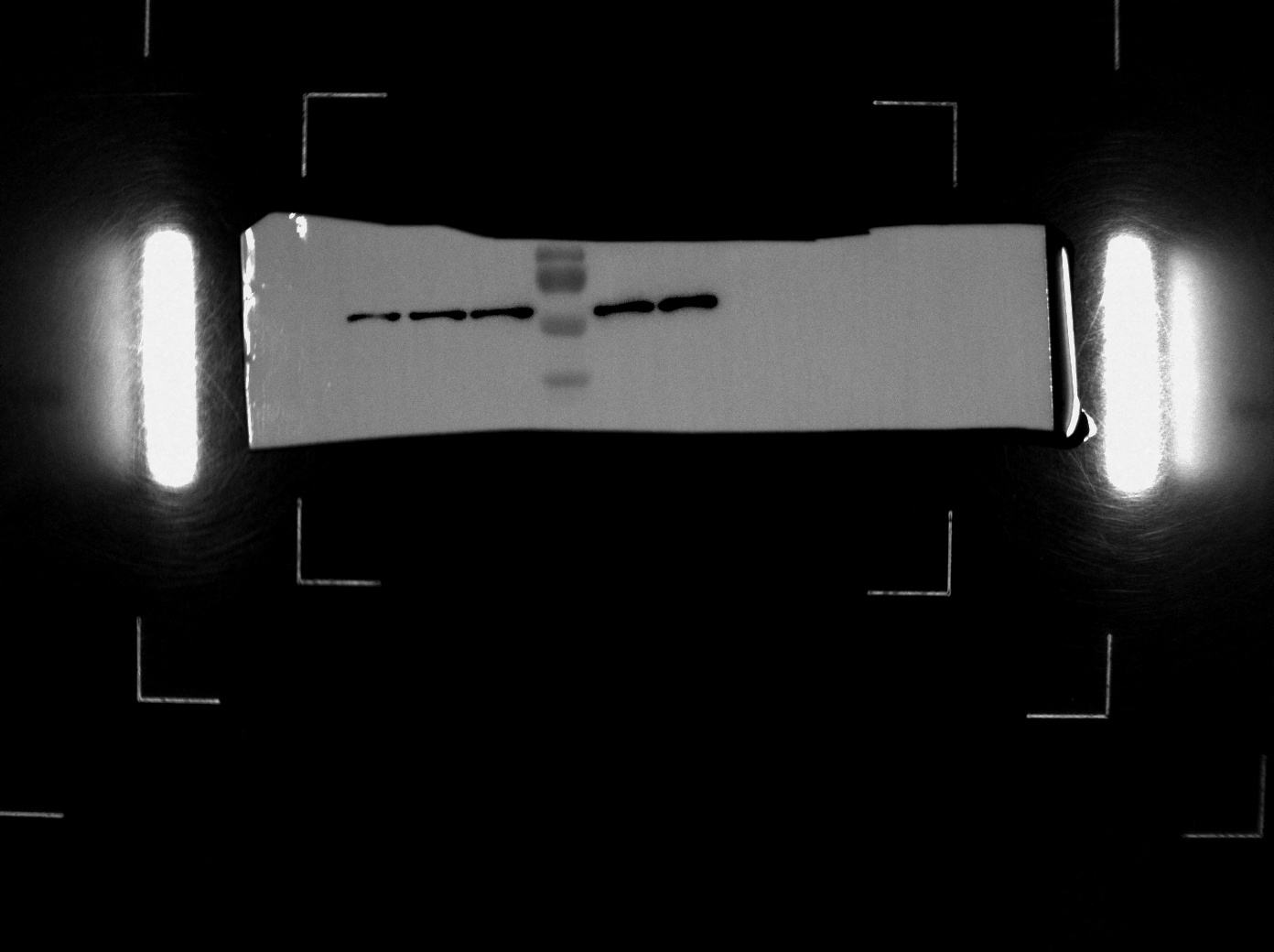

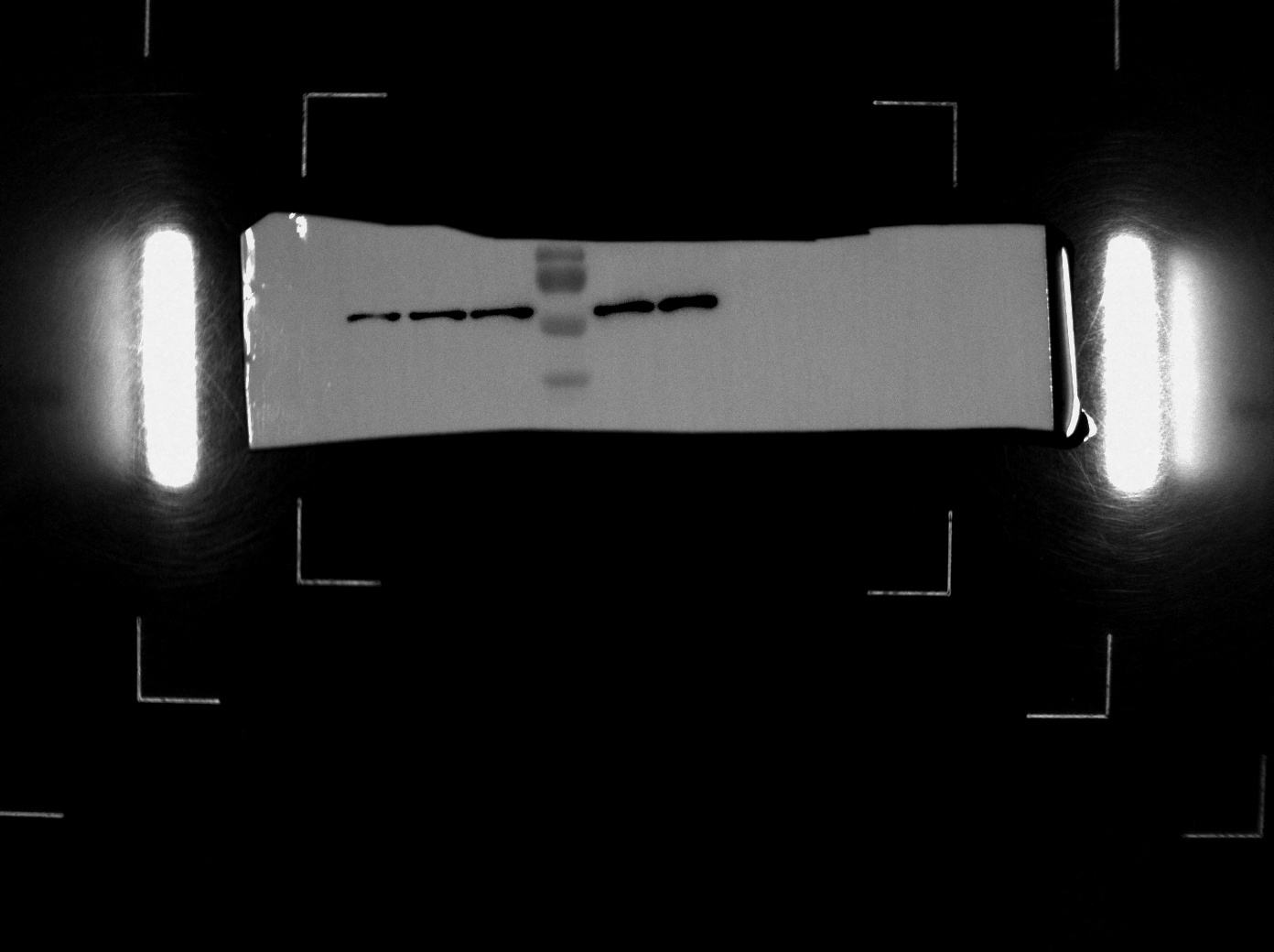


**Figure S2.** The stable expression of the protein of interest identified by western blot detection. The left figure is original images for Blots, and the right figure was applied in the article. 1-3, 5-6 lines represent *L. casei* pPG-Alr-COE/△*Alr W56* strains 10th to 50th generation protein expression; 4 represent the protein marker; 7 represent the strain pPG-T7g10-PPT/△*Alr W56*; 8 represent the strain △*Alr W56.* The densitometry readings/intensity ratio of band from left to right were 223.28, 232.01, 234.95, 237.94 and 241.31.
